# COVID-19 pandemic fatigue and its sociodemographic and psycho-behavioral correlates: a population-based cross-sectional study in Hong Kong

**DOI:** 10.1038/s41598-022-19692-6

**Published:** 2022-09-27

**Authors:** Hiu Tin Leung, Wei-Jie Gong, Shirley M. M. Sit, Agnes Y. K. Lai, Sai Yin Ho, Man Ping Wang, Tai Hing Lam

**Affiliations:** 1 School of Public Health, The University of Hong Kong, Hong Kong, China; 2 Department of General Practice, Health Science Center, Shenzhen University, Guangdong, China; 3 School of Nursing, The University of Hong Kong, Hong Kong, China

**Keywords:** Public health, Population screening

## Abstract

Pandemic fatigue is a growing public health concern of the lingering COVID-19 pandemic. Despite its widespread mass media coverage, systematic empirical investigations are scarce. Under the Hong Kong Jockey Club SMART Family-Link Project, we conducted online and telephone surveys amid the pandemic in February to March 2021 to assess self-reported pandemic fatigue (range 0–10) in Hong Kong adults (*N* = 4726) and its associations with sociodemographic and psycho-behavioral (high vs low to moderate) variables. Data were weighted by sex, age, and education of the general population. Binary logistic regression models yielded adjusted odds ratios (aORs) for high pandemic fatigue (score ≥ 7) for sociodemographic and psycho-behavioral variables. 43.7% reported high pandemic fatigue. It was less common in older people (55–64 years: aOR 0.56, 95% CI 0.39–0.82; 65 + years: 0.33, 0.21–0.52) versus age group 18–24 years, but more common in those with tertiary education (1.36, 1.15–1.62) versus secondary or below. High pandemic fatigue was positively associated with depressive symptoms (aOR 1.83, 95% CI 1.55–2.17), anxiety symptoms (1.87, 1.58–2.20), loneliness (1.75, 1.32–2.31), personal fear of COVID-19 (2.61, 2.12–3.23), family fear of COVID-19 (2.03, 1.67–2.47), and current alcohol use (1.16, 1.00–1.33), but negatively associated with self-rated health (0.79, 0.68–0.92), personal happiness (0.63, 0.55–0.72), personal adversity coping capability (0.71, 0.63–0.81), family adversity coping capability (0.79, 0.69–0.90), family well-being (0.84, 0.73–0.97), family communication quality (0.86, 0.75–0.98), and frequent home exercise (0.82, 0.69–0.96; versus less frequent). We first used a single-item tool to measure COVID-19 pandemic fatigue, showing that it was common and associated with worse mental health, lower levels of personal and family well-being and alcohol use.

## Introduction

The lingering coronavirus disease (COVID-19) pandemic and corresponding public health measures have induced psychosocial distress globally and in Hong Kong, one of the most developed cities in China^1,2^. Beginning in mid-2020, although with no lockdown, there have been increasing media reports that the general public was experiencing burnout and fatigue-like symptoms such as feeling tired, worried and depressed from prolonged enforcement of stringent pandemic measures. According to the World Health Organization (WHO), pandemic fatigue is defined as “demotivation to follow recommended protective behaviors, emerging gradually over time and affected by a number of emotions, experiences and perceptions”^3^. As public adherence to protective behaviors is critical to containing the virus, pandemic fatigue poses a serious public health concern amid the COVID-19 pandemic^3^.

As one of the biggest global health threats in recent history, COVID-19 would naturally cause stress and adaptive responses in our physical and psychological systems. Exposure to non-specific stress is known to evokes an integrated syndrome of closely inter-related adaptive reactions^3–5^. Initial symptoms resemble the “fight-or-flight” response pattern. However, this cannot continue indefinitely and exhaustion may set in, triggering fatigue, depression, and anxiety^4,5^. Drawing on the COM-B model, the WHO posits that pandemic fatigue is influenced by multiple factors involving individual motivation (e.g. trust, risk perception), individual capability (e.g. awareness, self-efficacy), and contextual opportunity (e.g. regulations, social norms)^3^.

Pandemic fatigue was variously described in the literature as: (1) a subjective feeling associated with depression, anxiety, lower resilience, poorer sleep quality, and poorer mental health among healthcare workers and patients amid the prolonged pandemic^6–11^, (2) lower adherence to certain COVID-19 protective behaviors over time in the general population^9,11–16^, or (3) an explanatory construct for the underlying phenomena^17,18^. A concept closely related to pandemic fatigue is that of “pandemic burnout”, a state of feeling emotionally and physically drained from prolonged anti-pandemic measures^19^. It could be measured by a short, five-item instrument, and was positively correlated with fear of COVID-19 and intention to adopt a “living with COVID” policy, but negatively correlated with COVID-19 vaccination status and support for the “dynamic zero COVID-19 strategy” in a convenient Hong Kong sample^19^.

However, still lacking in the literature is a simple, single-item tool to specifically measure “pandemic fatigue” (as generally understood but distinct from “pandemic burnout”) in a representative sample of the general population and how it relates to a wider range of other better understood psycho-behavioral constructs. In Hong Kong or mainland China, we found no peer-reviewed publications specifically on “pandemic fatigue” as a self-reported subjective feeling, although usage of the term by the mass media has become widespread and popular. Moreover, the fourth wave outbreak in Hong Kong (from November 2020 to March 2021) has been attributed to pandemic fatigue, with lapses in social distancing and mask-wearing resulting in singing and dancing clusters with over 730 confirmed cases in November 2020^20,21^.

Since then, Hong Kong has successfully controlled COVID-19 outbreaks using an elimination strategy with no lockdown but zero and almost zero local cases for nine months since the fourth wave by universal masking (voluntary with almost 100% adherence for over two years followed by mandatory requirement to date), stringent social distancing, social gathering ban, intensive contact tracing, large-scale testing, strict isolation and quarantine, border control and travel restrictions, with strong enforcement and almost complete population compliance but no lockdown^22,23^. Since February 2022, Hong Kong has been hit by a fifth wave outbreak which has persisted for a month or so, and media reports of pandemic fatigue has increased again recently.

Under the Hong Kong Jockey Club SMART Family-Link Project, we conducted a second Family Amidst COVID-19 (FamCov2) survey in February 2021, with the aim to study the personal and family wellbeing in the Hong Kong general population amid the COVID-19 pandemic, shortly after the fourth wave outbreak in Hong Kong was under control^24^. The present study examined the prevalence of self-reported pandemic fatigue and its associations with sociodemographic and psycho-behavioral variables.

## Materials and methods

### Sample and procedures

FamCov2 was a population-based cross-sectional dual sampling telephone and online survey conducted from 22 February to 23 March 2021^24^. The target population was Cantonese-speaking Hong Kong residents aged 18 years or above, who had landline or mobile telephone numbers or email accounts. The questionnaire (in Chinese) was developed by our project team, based on that of FamCov1^25,26^. The Hong Kong Public Opinion Research Institute (HKPORI), a well-known local survey agency, was commissioned to conduct the survey. HKPORI reviewed the questionnaire and tested it in a pilot survey on 5 November 2020 with 20 participants from the target population (including 10 landline and 10 mobile numbers). Overall, the respondents found the questions easy to understand and answer so only minor changes to the wordings and options were made. Some less important questions were also dropped to shorten the interview.

Random telephone interviews were conducted by closely monitored interviewers. All data were collected using HKPORI’s Web-based Computer Assisted Telephone Interviewing (Web-CATI) system that allows real-time data capture and consolidation. In both the landline and mobile telephone surveys, the numbers were randomly generated using known prefixes assigned to telecommunication service providers under the Numbering Plan of the Office of the Communications Authority (OFCA). Non-working numbers were identified by the computer system that can detect tritone signal and manual dialing records. Confirmed invalid numbers were eliminated and the remaining numbers mixed in random order to produce the final telephone sample. For landline numbers, as a second-level sampling, when telephone contact was successfully established with a target household, only one eligible respondent was selected from all those present using the “next birthday rule”. No second-level sampling was done for the mobile sample. Each interview took about 25 min. Among the 2420 valid telephone numbers sampled, 1522 respondents (62.9%) completed the interview.

The online questionnaire was anonymous and self-administered. All data were collected in an e-platform. The methods were reported previously^25,26^. Briefly, invitations by e-mail, with an access link to the survey website, were sent to HKPORI's probability and non-probability based PopPanel numbers aged 18 or above by HKPORI. The target respondents were given information on the purpose of the survey, with an emphasis on confidentiality. Email invitation was sent to 95,705 adults with valid email addresses from the panel. 48,825 (51.0%) invitation emails were opened, and 6013 respondents (12.3% of 48,825) successfully completed the survey.

Ethics approval was obtained from the Institutional Review Board of the University of Hong Kong/Hospital Authority Hong Kong West Cluster (IRB reference no.: UW20-651). Informed consent was obtained from all subjects and/or their legal guardians. All methods were performed in accordance with the relevant guidelines and regulations.

### Public involvement

The members of the public who responded to our surveys were not possible to be involved in the design, or conduct, or reporting, or dissemination plans of our research as the questionnaire was anonymous. We will seek to disseminate the main results of this research to the public.

### Measures

As our literature review has shown that the term “ “pandemic fatigue” is a popular term widely used in the mass media in Hong Kong since mid-2020, we assessed pandemic fatigue by a single direct question, “Do you think you have pandemic fatigue?” on a scale of 0 (not at all) to 10 (very fatigued). Self-rated health was assessed by “Overall, how do you think your health is?” on a scale of 1 (poor) to 5 (extremely good). Personal happiness was assessed by “How happy do you think you are?” on a scale of 0 (not at all happy) to 10 (very happy)^27^. Personal adversity coping capability was assessed by “How do you rate your capability to cope with adversities?” on a scale of 0 (not at all capable) to 10 (very capable). Family adversity coping capability was assessed by “How do you rate your family’s capability to cope with adversities?” on a scale of 0 (not at all capable) to 10 (very capable)^24^. Family well-being was assessed by the mean score to three questions: “How healthy/happy/harmonious (3 separate questions) do you think your family is?” on a scale of 0 (not at all healthy/happy/harmonious) to 10 (very healthy/happy/harmonious)^27,28^. Family communication quality was assessed by “How do you find the quality of communication between you and your family members?” on a scale of 0 (very poor) to 10 (very good)^29^. Personal fear of COVID-19 was assessed by “Has COVID-19 caused you fear?” on a scale of 0 (no fear at all) to 10 (very fearful). Family fear of COVID-19 was assessed by “Has COVID-19 caused your family fear?” on a scale of 0 (no fear at all) to 10 (very fearful)^26^. Knowledge on COVID-19 was assessed by “How much knowledge do you have on COVID-19 and anti-pandemic practices?” on a scale of 0 (no knowledge at all) to 10 (very knowledgeable). Confidence to cope with COVID-19 was assessed by “How much confidence do you have in coping with the COVID-19 pandemic?” on a scale of 0 (not at all confident) to 10 (very confident). Loneliness was assessed by “Over the past 7 days, how often did you feel lonely?”. Frequency of home exercise was assessed by “Over the past 7 days, how often did you exercise at home?”. Depressive and anxiety symptoms were assessed separately using the four-item Patient Health Questionnaire (PHQ-4)^30,31^. Smoking was assessed by “Do you have a smoking (including cigarettes, electronic cigarettes and heated tobacco products) habit?” and alcohol use was assessed by “How often do you consume alcoholic drinks (e.g. beer, wine, spirits)?” Both questions were analyzed as current versus never or former. Sex, age, educational attainment, employment status, household income, and housing type were also asked.

We dichotomized pandemic fatigue and psycho-behavioral variables as “high” versus “low to moderate” in analyses. The distribution of pandemic fatigue scores showed a double-peak occurring at 5 and 7 (Supplementary Table 1) and therefore scores of 7 or above were defined as high for pandemic fatigue. For consistency, scores of 7 or above were also defined as high for personal happiness, personal adversity coping capability, family well-being, family communication quality, family adversity coping capability, personal fear of COVID-19, family fear of COVID-19, knowledge on COVID-19, and confidence to cope with COVID-19. Self-rated health was defined as high for scores of 4 or above. Loneliness was defined as high and home exercise was defined as frequent (versus less frequent) for 5 days or more per week. For PHQ-4, depressive symptoms were defined as high for scores of 3 or above on the depression subscale, as were anxiety symptoms on the anxiety subscale^30,31^.

Sociodemographic variables were coded as follows: age into six groups (18–24, 25–34, 35–44, 45–54, 55–64, 65 years or above), education into two groups (secondary or lower, tertiary), employment into five groups (full-time, students, home-makers, retired, unemployed), household income into three groups (HKD 19,999 or below, HKD 20000–39999, HKD 40,000 or above), and housing into two groups (rented, owned).

### Statistical analysis

All statistical analyses were conducted using IBM SPSS version 26. Statistical significance was determined by *P* < 0.05. To improve the representativeness of the sample, all data were weighted using random iterative method (RIM) weighting based on sex, age, and education distribution of the Hong Kong general adult population in 2020^32^. Binary logistic regression models were used to examine the association of pandemic fatigue with sociodemographic and psycho-behavioral variables. Associations between high pandemic fatigue and all 16 of the above dichotomized psycho-behavioral variables were estimated by crude and adjusted odd ratios (ORs) with 95% confidence intervals (CIs), with the latter adjusting for all sociodemographic variables.

## Results

### Participants characteristics and associations with high pandemic fatigue

Table 1 shows in the weighted sample 52.4% were female, 79.9% were aged 18–64 years, 35.3% had tertiary education, 55.2% had full-time employment, and 31.0% had a monthly household income of HK$19,999 or lower (US$1 = HK$7.8). The weighted prevalence of high pandemic fatigue was 43.7% (95% CI 41.2%− 44.3%) overall (Supplementary Table 1 shows the pandemic fatigue scores approximate a normal distribution). Adjusted odds ratios (aORs) of high pandemic fatigue were reduced for age 55–64 (aOR 0.56, *P* < 0.01) and age 65 or above (aOR 0.33, *P* < 0.001) versus age 18–24, and elevated for tertiary education (aOR 1.36, *P* < 0.01) versus secondary or below. No other sociodemographic factors showed significant associations after mutual adjustment.

**Table 1:** Sociodemographic characteristics of the sample and their associations with high pandemic fatigue.

Variables	Unweighted n (%)	Weighted n (%)	Weighted n (%) of high pandemic fatigue	Binary logistic regression models	
				Crude OR	Adjusted OR
				(95% CI)	(95% CI)^b^
Overall	4726 (100)	4726 (100)	2051 (43.7)	–	–
**Sex**					
Male	2264 (48.2)	2237 (47.6)	934 (42.0)	Reference	Reference
Female	2432 (51.8)	2459 (52.4)	1105 (45.2)	1.08 (0.96–1.21)	1.14 (1.00–1.30)
**Age group (years)**					
18–24	378 (8.1)	405 (8.6)	194 (48.3)	Reference	Reference
25–34	895 (19.1)	717 (15.3)	334 (46.6)	1.11 (0.87–1.41)	1.06 (0.75–1.49)
35–44	1001 (21.3)	805 (17.1)	417 (52.1)	1.30 (1.02–1.64)*	1.21 (0.86–1.71)
45–54	984 (21.0)	879 (18.7)	426 (48.6)	1.01 (0.80–1.29)	0.91 (0.64–1.29)
55–64	832 (17.6)	949 (20.2)	346 (36.7)	0.59 (0.46–0.76)***	0.56 (0.39–0.82)**
65 or above	603 (12.8)	940 (20.0)	323 (34.8)	0.31 (0.24–0.41)***	0.33 (0.21–0.52)***
**Education**					
Secondary or below	1204 (25.8)	3020 (64.7)	1258 (42.0)	Reference	Reference
Tertiary	3462 (74.2)	1650 (35.3)	770 (46.8)	1.91 (1.66–2.18)***	1.36 (1.15–1.62)***
**Employment**					
Full-time	3033 (65.6)	2552 (55.2)	1183 (46.5)	Reference	Reference
Students	244 (5.3)	265 (5.7)	123 (46.6)	0.85 (0.65–1.10)	0.78 (0.53–1.17)
Home-makers	318 (6.9)	421 (9.1)	194 (46.4)	0.59 (0.47–0.75)***	0.99 (0.74–1.33)
Retired	779 (16.8)	1096 (23.7)	390 (35.9)	0.41 (0.34–0.48)***	0.95 (0.73–1.25)
Unemployed	253 (5.5)	292 (6.3)	118 (41.1)	0.86 (0.66–1.11)	0.96 (0.72–1.29)
**Household income (HK$)**^**a**^					
$19,999 or below	924 (22.9)	1275 (31.0)	511 (40.7)	Reference	Reference
$20,000 – 39,999	1029 (25.5)	1333 (32.4)	592 (44.5)	1.50 (1.25–1.79)***	1.08 (0.89–1.31)
$40,000 or above	2082 (51.6)	1507 (36.6)	684 (45.4)	1.53 (1.31–1.80)***	1.00 (0.83–1.21)
**Housing**					
Rented	1738 (37.2)	1834 (39.2)	800 (44.0)	Reference	Reference
Owned	2938 (62.8)	2846 (60.8)	1232 (43.5)	0.97 (0.86–1.09)	1.04 (0.90–1.89)

*a*US$1 = HK$7.8.

*b*OR adjusted for all sociodemographic variables.

Missing data were excluded. Weighting was applied based on the distribution of sex, age, and education in the Hong Kong population 2020 census data. *P < 0.05, **P < 0.01 ***P < 0.001.

### Psycho-behavioral variables associated with high pandemic fatigue

Table 2 shows, with adjustment, high pandemic fatigue was positively associated with high loneliness (aOR 1.75, *P* < 0.001), high depressive symptoms (aOR 1.83, *P* < 0.001), high anxiety symptoms (aOR 1.87, *P* < 0.001), high personal fear of COVID-19 (aOR 2.61, *P* < 0.001), and high family fear of COVID-19 (aOR 2.03, *P* < 0.001), and negatively associated with high self-rated health (aOR 0.79, *P* < 0.01), high personal happiness (aOR 0.63, *P *< 0.001), high personal adversity coping capability (aOR 0.71, *P* < 0.001), high family adversity coping capability (aOR 0.79, *P* < 0.001), high family well-being (aOR 0.84, *P* < 0.05), and high family communication quality (aOR 0.86, *P* < 0.05). High self-rated knowledge on COVID-19 (aOR 1.33, *P* < 0.001) showed positive associations with high pandemic fatigue, whereas high confidence to cope with COVID-19 (aOR 0.96, *P* > 0.05) showed no significant association. Finally, frequent home exercise (aOR 0.82, *P* < 0.05) was negatively associated with high pandemic fatigue, whereas current alcohol use (aOR 1.16, *P* < 0.05) showed positive association.

**Table 2:** Associations between high pandemic fatigue and psycho-behavioral variables.

Variables	Weightedn (%)	Weighted n (%) of high pandemic fatigue	Binary logistic regression models	
			Crude OR (95% CI)	Adjusted OR (95% CI)^a^
**Self-rated health**				
Low to moderate (1–3)	3660 (77.7)	1642 (45.2)	Reference	Reference
High (≥ 4)	1052 (22.3)	396 (37.8)	0.84 (0.74–0.96)**	0.79 (0.68–0.92)**
**Personal happiness**				
Low to moderate (0–6)	2909 (61.6)	1421 (49.1)	Reference	Reference
High (≥ 7)	1810 (38.4)	624 (34.7)	0.61 (0.54–0.69)***	0.63 (0.55–0.72)***
**Personal adversity coping capability**				
Low to moderate (0–6)	2577 (55.0)	1206 (47.1)	Reference	Reference
High (≥ 7)	2112 (45.0)	832 (39.5)	0.72 (0.64–0.81)***	0.71 (0.63–0.81)***
**Family adversity coping capability**				
Low to moderate (0–6)	2206 (46.7)	1018 (46.5)	Reference	Reference
High (≥ 7)	2475 (52.4)	1015 (41.2)	0.75 (0.67–0.84)***	0.79 (0.69–0.90)***
**Family well-being**				
Low to moderate (0–6)	1805 (38.2)	824 (45.9)	Reference	Reference
High (≥ 7)	2358 (56.6)	981 (41.7)	0.77 (0.68–0.87)***	0.84 (0.73–0.97)*
**Family communication quality**				
Low to moderate (0–6)	2449 (51.8)	1112 (45.6)	Reference	Reference
High (≥ 7)	2271 (48.0)	937 (41.5)	0.77 (0.68–0.86)***	0.86 (0.75–0.98)*
**Loneliness**				
Low to moderate (0–4 days/week)	4285 (93.6)	1818 (42.7)	Reference	Reference
High (≥ 5 days/week)	295 (6.4)	174 (59.4)	1.74 (1.37–2.20)***	1.75 (1.32–2.31)***
**Depressive symptoms**				
Low to moderate (0–2)	3833 (81.8)	1545 (40.6)	Reference	Reference
High (≥ 3)	854 (18.2)	490 (57.5)	1.95 (1.69–2.27)***	1.83 (1.55–2.17)***
**Anxiety symptoms**				
Low to moderate (0–2)	3784 (80.9)	1493 (39.7)	Reference	Reference
High (≥ 3)	896 (19.1)	539 (60.4)	2.06 (1.78–2.38)***	1.87 (1.58–2.20)***
**Personal fear of COVID-19**				
Low to moderate (0–6)	1630 (71.4)	571 (35.1)	Reference	Reference
High (≥ 7)	652 (28.6)	379 (58.7)	2.75 (2.27–3.31)***	2.61 (2.12–3.23)***
**Family fear of COVID-19**				
Low to moderate (0–6)	1506 (67.6)	530 (35.3)	Reference	Reference
High (≥ 7)	723 (32.4)	406 (56.3)	2.25 (1.89–2.69)***	2.03 (1.67–2.47)***
**Self-rated knowledge on COVID-19**				
Low to moderate (0–6)	1348 (28.6)	495 (37.0)	Reference	Reference
High (≥ 7)	3363 (71.4)	1551 (46.4)	1.49 (1.31–1.70)***	1.33 (1.15–1.54)***
**Confidence to cope with COVID-19**				
Low to moderate (0–6)	1905 (41.0)	814 (42.8)	Reference	Reference
High (≥ 7)	2737 (59.0)	1212 (44.4)	1.02 (0.90–1.15)	0.96 (0.84–1.09)
**Home exercise**				
Less frequent (0–4 days/week)	3488 (73.8)	1591 (45.7)	Reference	Reference
Frequent (≥ 5 days/week)	1214 (25.8)	451 (37.6)	0.57 (0.50–0.66)***	0.82 (0.69–0.96)*
**Smoking**				
Never or former	4163 (88.6)	1765 (42.6)	Reference	Reference
Current	535 (11.4)	274 (51.7)	1.27 (1.02–1.68)*	1.23 (0.97–1.58)
**Alcohol use**				
Never or former	1703 (36.4)	673 (39.7)	Reference	Reference
Current	2973 (63.6)	1360 (46.1)	1.40 (1.24–1.59)***	1.16 (1.00–1.33)*

*a*. OR adjusted for all sociodemographic variables.

Missing data were excluded. Weighting was applied based on the distribution of sex, age, and education in the Hong Kong population 2020 census data. *P < 0.05, **P < 0.01 ***P < 0.001.

## Discussion

Our study was the first to use a single-item tool to measure pandemic fatigue. We showed that it was associated with sociodemographic characteristics (younger age, tertiary education) and psycho-behavioral variables in a population survey amid the COVID-19 pandemic. The directions of most associations were as expected: negative for positive outcomes of self-rated health, personal happiness, personal and family adversity coping capability, family well-being, family communication quality, and home exercise, and positive for negative outcomes of depressive and anxiety symptoms, loneliness, personal and family fear of COVID-19, and alcohol use.

The associations identified above provide some much-needed evidence for the construct validity for pandemic fatigue in the general population. It has been argued that pandemic fatigue is merely a vaguely defined popular term, suggesting that it is a putative construct that has little scientific or empirical basis in the current literature^33^. However, by using an original one-item tool, we have provided data on the normal distribution of pandemic fatigue score and prevalence of high pandemic fatigue (about 44%) defined by a score of 7 to 10. The term is commonly used and should be well understood by most people in Hong Kong. Almost all (99.4%) of our respondents answered the question, and only 5% answered “none at all”, indicating such feeling or perception was widespread in the population. We have further shown that high pandemic fatigue was associated with many well-understood psychological and behavioral factors as expected.

In line with two recent reports^34,35^, we found pandemic fatigue more common in young people. The preventive measures might have had a heavier burden on young people typically involved in work, study and a more active lifestyle. In Hong Kong, older people had experienced the 2003 SARS outbreak with higher case fatality rates and their past coping experience could also explain their lower pandemic fatigue. We found pandemic fatigue associated with tertiary education. Those with tertiary education should be more knowledgeable, and thus was consistent with another unexpected result in the present study that more people who reported having more knowledge of COVID-19 also reported high pandemic fatigue. We previously reported that frequent use of the Internet as a source of COVID-19-related information was associated with psychological distress symptoms^36^, and that health information seeking was more prevalent among younger adults and those with higher education attainment^37^. These findings corroborate with the present results in suggesting that increased exposure to COVID-19-related information, especially among the younger and more educated people, could lead to earlier and stricter compliance of stringent control measures, which when prolonged with uncertainty about when the pandemic would end would lead to earlier and higher pandemic fatigue and related adverse health effects.

The expected associations between pandemic fatigue and depressive and anxiety symptoms from PHQ-4, a widely used validated ultra-brief scale^30,31^, support the validity of our one-item scale and highlight the significance of this issue on population mental health. We have previously reported an increase in depression and anxiety in the general population amid the COVID-19 pandemic in Hong Kong^1,2^. The present study also found that both personal and family fear of COVID-19 were most strongly associated with high pandemic fatigue, and seemed to be more so than depression and anxiety. This echoes with previous work on “pandemic burnout”, which showed that this related construct was also positively correlated with fear of COVID-19^19^. As we have also shown in FamCov1 that fear of COVID-19 was associated positively with perceived harms and negatively with perceived benefits from the pandemic^25,26^, further studies on whether high pandemic fatigue show similar associations like fear of COVID-19 are warranted.

On the other hand, we found several positive personal and family factors or outcomes were negatively related to pandemic fatigue. Those with high pandemic fatigue had lower personal happiness, personal and family adversity coping capability, family well-being, and family communication quality. These were as expected and consistent with the above results. We also found that loneliness, also measured by a simple direct question, was associated with high pandemic fatigue, and such results mutually support the validity of simple direct tools. Maintaining social contact could reduce loneliness and be important for mental well-being amid the pandemic. Evidence in the literature suggest an integral family is important in maintaining basic physical and mental health and well-being under ordinary pre-COVID-19 circumstances^38^. Stronger family cohesion and better parent–child relationships are also associated with better life satisfaction, health, and behavioral outcomes^39^. In the face of adversity, such as the COVID-19 pandemic, family relationships could also function as a buffer against external stressors and various risks of adverse events^40^. On the other hand, whether social distancing leading to loneliness could lead to pandemic fatigue and lower compliance with stringent COVID-19 control measure should be further studied.

In addition, the present study has shown that a few behavioral variables were also associated with high pandemic fatigue, negatively with frequent home exercise and positively with alcohol use (but the increased OR for smoking was not significant after adjustment). It is well established that exercise can help alleviate depression^41^. Alcohol consumption was associated with depressive and anxiety symptoms amid the pandemic^42,43^. Our results suggest that pandemic fatigue could have adverse effects on lifestyle factors and related behavioral health and well-being.

A limitation of the present study was that we did not examine whether pandemic fatigue was associated with reduced motivation to adopt protective or infection control behaviors during the prolonged pandemic^3^. In a cross-country study comparing China and Poland, which encouraged and discouraged face mask, respective, face masking was much more common in China (96.8% vs 35.0%) and was associated with a lower prevalence of physical symptoms and levels of anxiety, depression and stress^44^. However, the study was conducted during the early stage of the pandemic (January to March 2020) and further studies on pandemic fatigue are warranted. As our one-item question can be easily administered to a large sample of the general population and has shown some face and construct validity from its associations with many important psycho-behavioral variables as expected, future research on whether pandemic fatigue is associated with support of government pandemic control policies and adoption of infection control behaviors amid the pandemic, such as masking, hand sanitizing, and social distancing, is also warranted. Another limitation was that we made extensive use of one-item measures. While single-item questions offer some benefits, and that we have previously provided evidence for their validity^24,26–28^, one might criticize their use for lacking reliability measures. Further works are needed to establish certain forms of reliability (such as test–retest reliability) for our original, one-item questions^27^. Another limitation was that our cross-sectional data could be limited for causal inference. Finally, as our survey was conducted in Hong Kong, where compliance to control measures was good and the response rate was not high, it is uncertain whether our results could be generalized to other populations and contexts.

## Conclusions

Using a new, single-item tool, we found a high prevalence of pandemic fatigue (43.7%) in the general population of Hong Kong shortly after the fourth wave COVID-19 outbreak was brought under control using an elimination strategy. However, there has been a fifth wave COVID-19 outbreak in Hong Kong since February 2022, and reports of pandemic fatigue has begun to re-emerge. We found high pandemic fatigue associated with worse mental health amid the COVID-19 pandemic, such as depressive and anxiety symptoms as well as fear of COVID-19. Moreover, it was negatively associated with some desirable personal and family factors or outcomes, such as personal happiness, family well-being, and adversity coping capability. It was also negatively associated with frequent home exercise but positively associated with alcohol use. Further studies on pandemic fatigue and whether interventions against such fatigue could enhance outbreak control, promote personal and family health and healthy lifestyle are urgently needed.

## Supplementary Information

Supplementary Information.

## Data Availability

The data that support the findings of this study are available from the corresponding author upon request.
