# Supplementary material for: COVID-19 pandemic fatigue and its sociodemographic and psycho-behavioral correlates: a population-based cross-sectional study in Hong Kong
Source: Sci Rep. 2022 Sep 27;12:16114. doi: 10.1038/s41598-022-19692-6 (PMC9514690; doi:10.1038/s41598-022-19692-6)
Supplement: Supplementary file 1 — Supplementary Information. [file 41598_2022_19692_MOESM1_ESM.docx]

**Supplementary Table 1**. Frequency distribution of pandemic fatigue scores

| **Variable** | **Unweighted n (%)** | **Weighted n (%)** |
| --- | --- | --- |
| Pandemic fatigue score  0 (Not at all)  1  2  3  4  5 (Half and half)  6  7  8  9  10 (Very fatigued) | 234 (5.0)  156 (3.3)  205 (4.4)  282 (6.0)  200 (4.3)  931 (19.8)  512 (10.9)  703 (15.0)  749 (15.9)  348 (7.4)  378 (8.0) | 216 (4.6)  199 (4.2)  198 (4.2)  310 (6.6)  195 (4.1)  1066 (22.7)  461 (9.8)  660 (14.1)  699 (14.9)  299 (6.4)  392 (8.4) |

Missing data were excluded. Weighting was applied based on the distribution of sex, age, and education in the Hong Kong population 2020 census data.
